# Case Report: *In situ* split liver transplantation from controlled donation after circulatory death donors assisted by normothermic regional perfusion: a case series

**DOI:** 10.3389/fimmu.2026.1933200

**Published:** 2026-09-22

**Authors:** Jisan Sun, Zhiwei Xiong, Haizhou Hao, Wentao Jiang

**Affiliations:** 1 Department of Liver Transplantation, First Central Hospital of Tianjin Medical University, Tianjin, China; 2 Tianjin Key Laboratory of Molecular Diagnosis and Treatment of Liver Cancer, Tianjin First Central Hospital, Tianjin, China; 3 Institute of Transplantation Medicine, Nankai University, Tianjin, China

**Keywords:** controlled donation after circulatory death, normothermic regional perfusion, outcomes, split liver transplantation, case series

## Abstract

**Introduction:**

Split liver transplantation (SLT) and controlled donation after circulatory death (cDCD) are two strategies used to expand the liver graft pool, but cDCD livers have traditionally been considered unsuitable for splitting because of warm ischemic injury, donor instability, and technical complexity. Normothermic regional perfusion (NRP) can restore oxygenated abdominal perfusion after death declaration and may create a physiologic window for *in situ* splitting.

**Case presentation:**

We report two cDCD donation procedures in which NRP supported *in situ* SLT. Following withdrawal of life-sustaining treatment and death declaration, NRP was initiated before hilar dissection and parenchymal transection. The livers were divided into left lateral segment and right trisegment grafts, which were transplanted into four recipients. Functional donor warm ischemia times were 15 and 20 min, and NRP durations were 154 and 235 min. During NRP, lactate levels decreased substantially in both donors (from 6.6 to 1.2 mmol/L and from 9.5 to 4.1 mmol/L, respectively), with concurrent pH normalization. All recipients had functioning grafts at last follow-up (17.2–20.9 months). One infant developed early allograft dysfunction in association with hepatic artery thrombosis and underwent successful revascularization. No recipient developed primary nonfunction, graft loss, or a clinically diagnosed biliary complication.

**Conclusion:**

This case series supports the technical feasibility of NRP-assisted *in situ* SLT from cDCD donors and documents encouraging intermediate-term graft function. The small sample does not establish safety or comparative effectiveness. Larger studies with standardized viability assessment and extended vascular and biliary follow-up are required.

## 1 Introduction

Liver transplantation remains the only curative treatment for many patients with end-stage liver disease, but the persistent mismatch between organ demand and donor supply continues to restrict access to transplantation (1). Donation after circulatory death (DCD) has therefore become an increasingly important complementary source of liver grafts, particularly in countries with mature controlled DCD (cDCD) programs (2, 3). Split liver transplantation (SLT) is another established strategy for expanding graft availability. By dividing one liver into two grafts, SLT can provide a left lateral segment for a pediatric recipient while preserving a right-sided graft for an adolescent or adult recipient. *In situ* splitting has potential advantages over ex vivo splitting because dissection is performed before cold storage, allowing better hemostatic control and avoiding additional bench-related cold ischemia (4, 5). However, SLT has generally been reserved for optimal donation after brain death (DBD) donors, and DCD grafts have remained a relative contraindication because of concerns regarding warm ischemic injury, hemodynamic instability, and biliary or vascular complications (6).

Normothermic regional perfusion (NRP), usually established with extracorporeal membrane oxygenation, restores warm oxygenated perfusion to abdominal organs after death declaration and can reduce the ischemic burden associated with conventional super-rapid recovery (7–9). Hemodynamic and metabolic trends during NRP may also inform graft acceptance, although no single parameter has been validated as a stand-alone viability criterion. In the present procedures, the perfused interval allowed hilar dissection and parenchymal transection before cold perfusion.

Previous reports have described *in situ* liver splitting under extracorporeal membrane oxygenation in a brain-dead donor and broader extracorporeal-support experience in DCD and DBD donation (10, 11). To our knowledge, *in situ* splitting of cDCD livers during abdominal NRP has not previously been reported. This setting differs from DBD-ECMO splitting because it requires circulatory determination of death, a no-touch interval, and exclusion of cerebral perfusion during NRP. We therefore describe the donation protocol, graft-assessment data, operative technique, and outcomes of two donors and four recipients.

## 2 Case presentation

### 2.1 Donation protocol, NRP assessment, and *in situ* splitting procedure

All donations were conducted under the Regulations on Human Organ Donation and Transplantation of the People’s Republic of China, effective 1 May 2024, and the Chinese Guidelines for Donation after Circulatory Determination of Death (2nd Edition). All donors were classified as Maastricht category III, and a multidisciplinary team confirmed the diagnosis of irreversible brain injury. The institutional protocol was approved by the Ethics Committee of Tianjin First Central Hospital (KYAP2025-159), and the donors’ families provided written informed consent. Intensive care physicians who were independent of the transplantation team determined death. After withdrawal of life-sustaining treatment (WLST), vital signs were monitored continuously. Circulatory arrest was defined by the absence of a palpable pulse and measurable blood pressure. A 5-min no-touch period was then observed while the absence of spontaneous circulation was confirmed. Total donor warm ischemia time (tDWIT) was defined from WLST to NRP initiation. Functional donor warm ischemia time (fDWIT) was defined from the first occurrence of mean arterial pressure below 60 mmHg or oxygen saturation below 80% to NRP initiation. After family consent and ethics approval, systemic heparinization, femoral arterial and venous cannulation, and placement of an intra-aortic balloon were performed before WLST. Following death declaration, the balloon was inflated at the thoracic aortic level to exclude cerebral circulation, and NRP was initiated through the pre-positioned femoral cannulas to perfuse the abdominal organs. The procedural sequence is shown in **Figure 1**.

**Figure 1:**
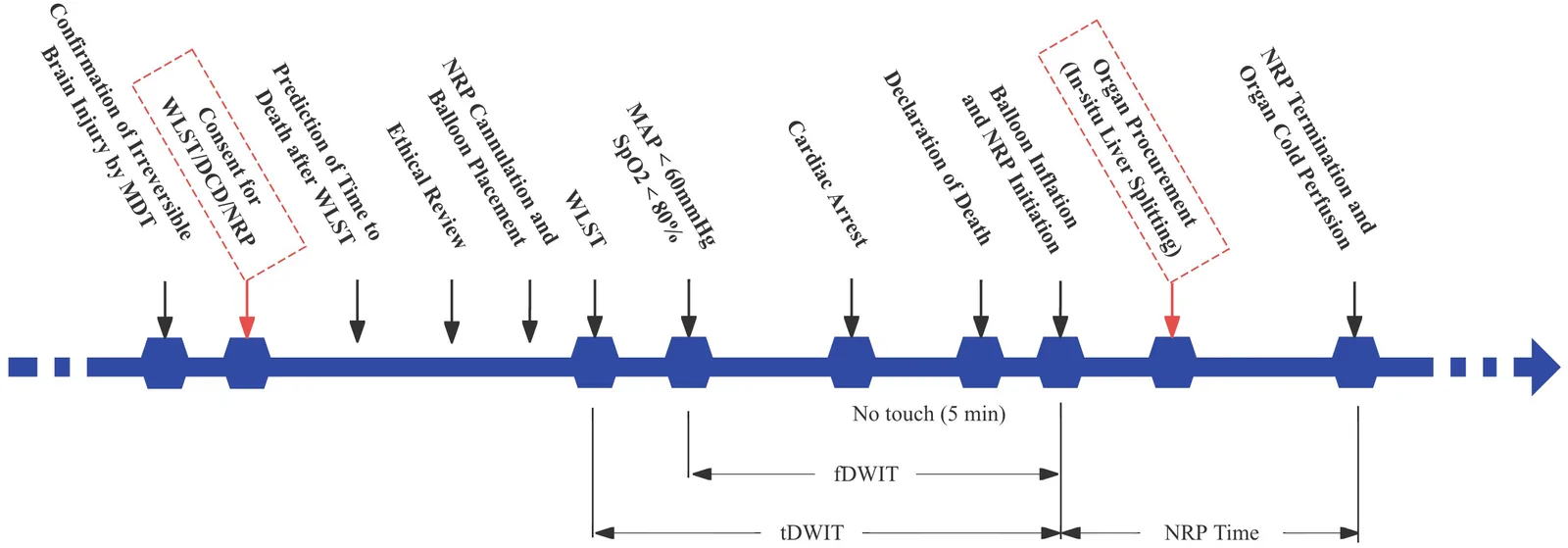
Organ donation and NRP-assisted *in situ* liver splitting workflow. DCD, donation after circulatory death; fDWIT, functional donor warm ischemia time; MAP, mean arterial pressure; NRP, normothermic regional perfusion; tDWIT, total donor warm ischemia time; WLST, withdrawal of life-sustaining treatment.

#### 2.1.1 NRP monitoring and graft acceptance

During NRP, circuit flow, temperature, blood-gas pH, lactate, and electrolytes were monitored. Graft acceptance was assessed from trends in lactate clearance and pH normalization, stable perfusion, macroscopic liver appearance and texture, and biopsy findings when available. Bile production was not quantified. Because only start and end metabolic measurements were available for this report, these observations were interpreted descriptively rather than as validated stand-alone viability criteria.

#### 2.1.2 *In situ* split liver procurement technique

Under continuous NRP, the liver was mobilized and the hepatic hilum was dissected systematically. The right hepatic artery, portal vein, and bile duct were preserved with the right trisegment graft, while the left hepatic artery and portal-vein branches were dissected for the left lateral segment graft. Intraoperative ultrasonography guided parenchymal transection along a plane immediately to the right of the falciform ligament, ligating or clipping encountered vascular and biliary branches. After completion of the split, cold *in situ* perfusion was performed through the portal vein and hepatic artery using 4 °C University of Wisconsin solution, after which NRP was discontinued. The grafts were retrieved separately, preserved in cold University of Wisconsin solution, and underwent vascular and biliary reconstruction on the back table as required. Each liver yielded a left lateral segment graft (Couinaud segments II and III) and a right trisegment graft (segments I and IV-VIII). Size matching used the graft-to-recipient weight ratio (GRWR).

### 2.2 Immunosuppression, outcome definitions, and follow-up

Immunosuppression was individualized based on recipient age, body weight, renal function, and clinical course. Induction therapy comprised an interleukin-2 receptor antagonist (daclizumab or basiliximab) combined with intraoperative methylprednisolone. Postoperative maintenance was initiated with tacrolimus every 12 hours, with dosages adjusted to maintain target trough levels (5–10 ng/mL). Concurrently, methylprednisolone was tapered and transitioned to tacrolimus monotherapy to prevent acute rejection. Tacrolimus dosing was dynamically adjusted throughout the perioperative period and follow-up based on therapeutic drug monitoring. Specifically, Recipient 1 received intraoperative daclizumab 6 mg and methylprednisolone 60 mg, followed by tacrolimus 0.4 mg every 12 hours. Recipient 2 received daclizumab 35 mg and methylprednisolone 350 mg, followed by tacrolimus 1.5 mg every 12 hours. Recipient 3 received daclizumab 11 mg and methylprednisolone 110 mg, followed by tacrolimus 0.75 mg every 12 hours. Recipient 4 received basiliximab 20 mg and methylprednisolone 500 mg, followed by tacrolimus 2.5 mg every 12 hours. All doses were subsequently adjusted according to serial trough level monitoring and clinical response.

Early allograft dysfunction (EAD) was classified according to the Olthoff criteria as one or more of the following: total bilirubin at least 10 mg/dL on postoperative day (POD) 7, international normalized ratio at least 1.6 on POD 7, or alanine or aspartate aminotransferase above 2000 U/L within the first 7 postoperative days (12). Primary nonfunction (PNF) was defined as irreversible graft failure leading to retransplantation or death within 7 days. Acute rejection was recorded when biopsy-proven or treated clinically. Vascular patency was assessed by serial Doppler ultrasonography during admission and follow-up. Biliary complications included clinically or radiologically diagnosed leak, dilation, or stricture. Magnetic resonance or endoscopic retrograde cholangiography was performed only when clinically indicated. Graft survival was defined as a functioning graft without retransplantation, and recipient survival as being alive at last contact.

### 2.3 Donor 1 characteristics and recipient outcomes

Donor 1 was a 6-year-old boy with blood type O who was admitted with severe traumatic brain injury after a fall. He had no history of cardiopulmonary resuscitation. Head computed tomography showed cerebral hemorrhage with extensive subarachnoid hemorrhage. After five days without neurological improvement, the family consented to WLST and consented to liver and kidney donation. The tDWIT and fDWIT were 18 and 15 min, respectively, and NRP lasted 154 min. Mean NRP flow was 1.71 L/min. From NRP start to end, pH increased from 7.118 to 7.518 and lactate decreased from 6.6 to 1.2 mmol/L, corresponding to 81.8% clearance (**Figure 2**). The improving metabolic profile, stable perfusion, favorable liver appearance, and biopsy showing preserved architecture with only occasional focal hepatocyte necrosis supported graft acceptance.

**Figure 2:**
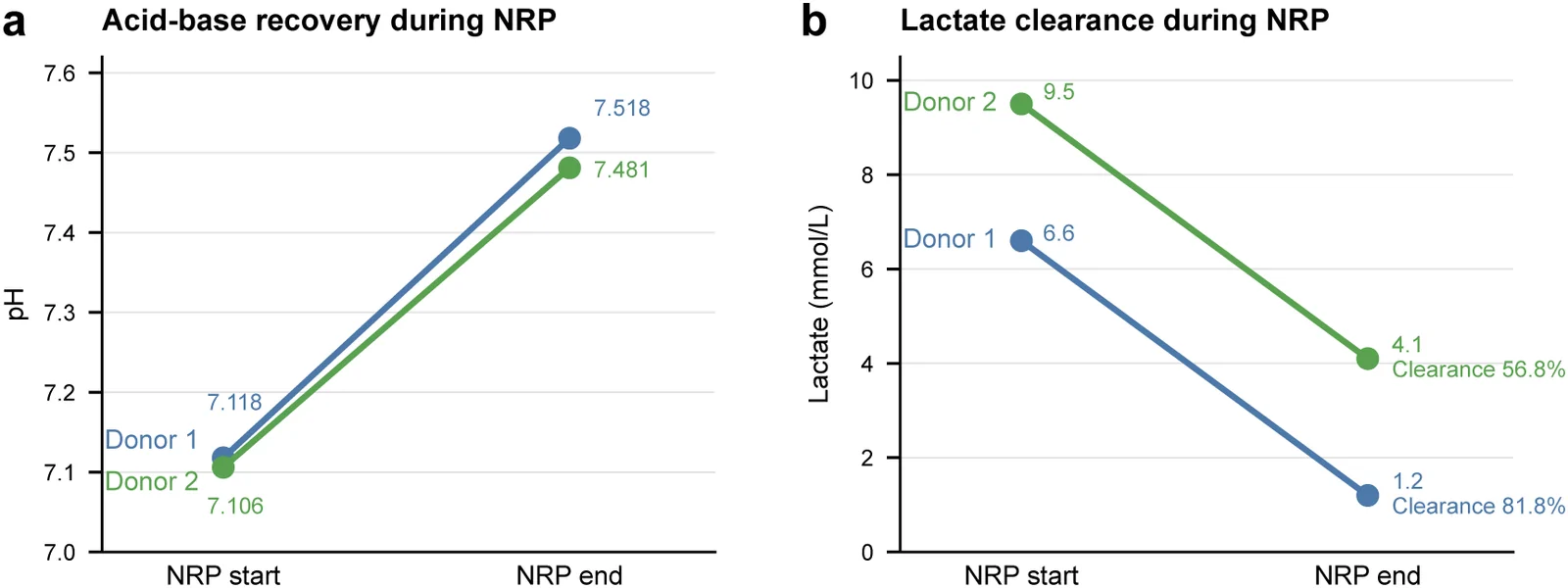
Metabolic changes during normothermic regional perfusion. Paired start-to-end measurements show pH normalization **(a)** and lactate reduction **(b)** in both donors.

Recipient 1: The 140-g left lateral segment graft (GRWR 2.55) was transplanted into a 5-month-old girl with blood type A and biliary atresia. The patient had undergone Kasai portoenterostomy at another hospital, but persistent jaundice led to transplantation. Her pretransplant PELD and Child scores were 36 and 8. Piggyback transplantation with biliary-enteric reconstruction was performed, and the cold ischemia time (CIT) was 269 min. On POD 1, Doppler and contrast-enhanced ultrasonography confirmed hepatic artery thrombosis (HAT). The small arterial diameter of the recipient was considered a likely technical contributor. Emergency laparotomy included thrombectomy, hepatic artery bypass reconstruction using the donor iliac artery interposition graft, and inferior vena cava thrombectomy. Postoperatively, argatroban was initiated as bridging therapy and transitioned to warfarin. Doppler ultrasonography on POD 7 showed restored hepatic arterial flow with a resistive index of 0.60. Recipient 1 met the EAD definition in association with HAT, with alanine aminotransferase reaching 3692.1 U/L on POD 2 and an INR of 1.69 on POD 7 (**Figure 3**). The postoperative peak total bilirubin of 252.9 µmol/L occurred on POD 4. Klebsiella oxytoca was cultured from abdominal drainage fluid on POD 12 and treated with meropenem. The patient was discharged on POD 30, with an INR of 1.87 while receiving oral warfarin anticoagulation. At the last assessment (20.8 months), both graft and recipient remained alive. Doppler ultrasonography confirmed patent hepatic arterial and portal venous flow, and no biliary dilation or clinically diagnosed stricture was documented.

**Figure 3:**
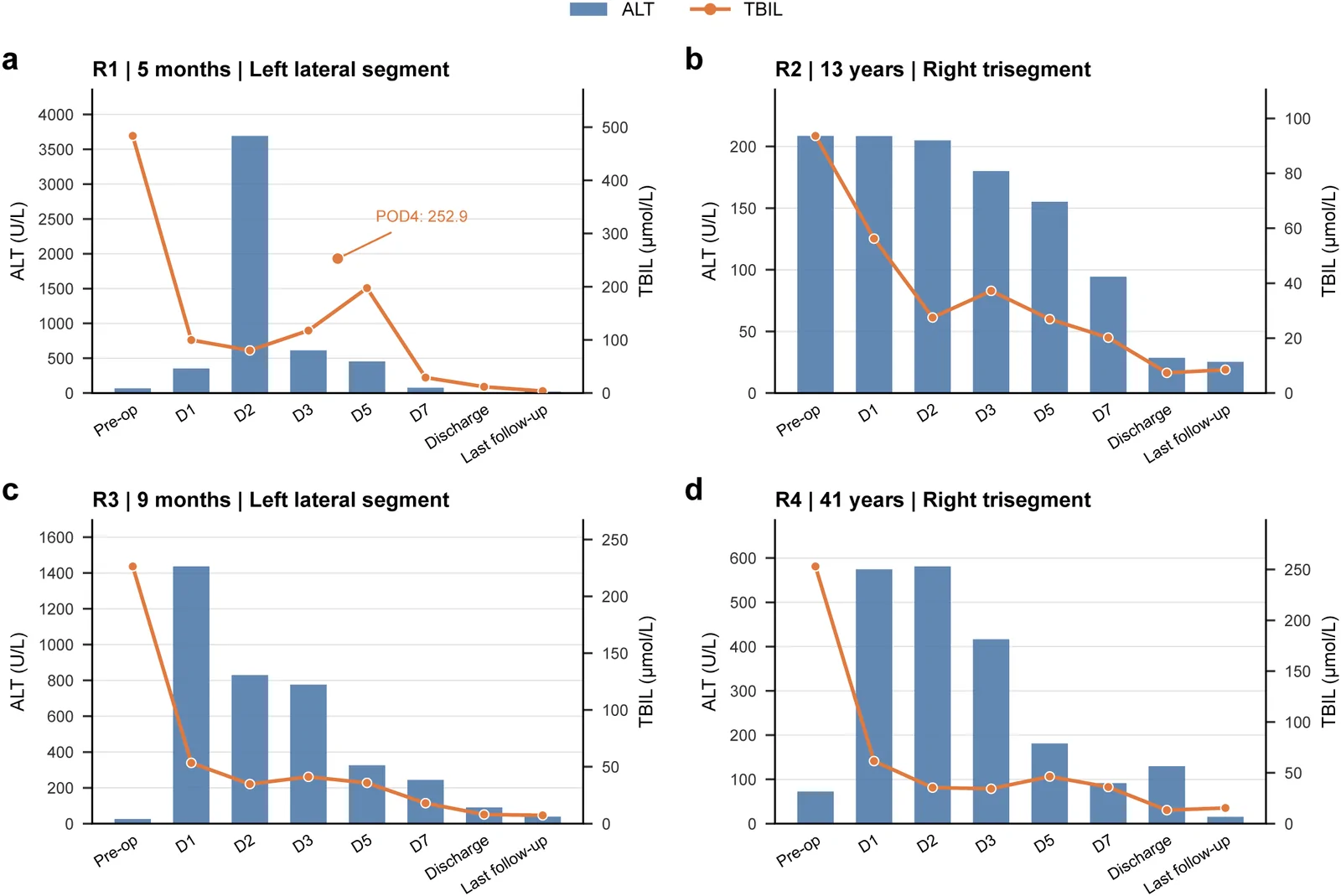
Per-recipient trajectories of alanine aminotransferase and total bilirubin. **(a–d)** Panels show Recipients 1–4 at eight standardized time points from pretransplant assessment to last follow-up. The clinically recorded TBIL peak of 252.9 µmol/L on POD 4 in R1 is annotated. ALT, alanine aminotransferase; D, day; Pre-op, pre-operative; R, recipient; TBIL, total bilirubin.

Recipient 2: The 510-g right trisegment graft (GRWR 1.46) was transplanted into a 13-year-old girl with blood type O and cryptogenic cirrhosis. Her pretransplant MELD and Child scores were 19 and 7. Piggyback transplantation with biliary-enteric reconstruction was performed, and the CIT was 160 min. Staphylococcus hominis was cultured from abdominal drainage fluid and treated with piperacillin-tazobactam (TZP). She left the intensive care unit on POD 2 and was discharged on POD 25. At 20.9 months, surveillance imaging demonstrated no biliary or vascular abnormalities, with both graft and recipient surviving.

### 2.4 Donor 2 characteristics and recipient outcomes

Donor 2 was a 37-year-old man with blood type A who experienced sudden cardiac arrest during exercise. The duration of cardiac arrest was unavailable, but spontaneous circulation returned after cardiopulmonary resuscitation. After nearly one month without neurological recovery, computed tomography suggested hypoxic-ischemic encephalopathy. The family elected WLST and consented to multi-organ donation. The tDWIT and fDWIT were 28 and 20 min, respectively, and NRP lasted 235 min. Mean NRP flow was 3.86 L/min. From NRP start to end, pH increased from 7.106 to 7.481 and lactate decreased from 9.5 to 4.1 mmol/L, corresponding to 56.8% clearance. Liver biopsy showed no significant abnormality.

Recipient 3: The 430-g left lateral segment graft (GRWR 3.91) was transplanted into a 9-month-old girl with blood type A and biliary atresia. At another hospital, the patient had undergone laparoscopic exploration and cholangiography, which confirmed biliary atresia, but Kasai portoenterostomy was not performed. Her PELD and Child scores were 17 and 9. Piggyback transplantation with biliary-enteric reconstruction was performed, and the CIT was 87 min. On POD 6, the platelet count fell to 5 × 10^9^/L without active bleeding. TZP-associated thrombocytopenia was suspected, and TZP was promptly discontinued while the tacrolimus-based immunosuppressive regimen was continued. The platelet count increased to 78 × 10^9^/L on POD 9 and exceeded 100 × 10^9^/L on POD 12, without recurrence or acute rejection. The patient was discharged on POD 18. At 17.2 months, the graft and recipient were alive, and there was no evidence of vascular occlusion, biliary dilation, or a clinically diagnosed stricture.

Recipient 4: The 1240-g right trisegment graft (GRWR 2.18) was transplanted into a 41-year-old man with blood type A, alcohol-related liver cirrhosis, and acute-on-chronic liver failure. Pretransplant MELD and Child scores were 19 and 13. Classic orthotopic transplantation with duct-to-duct biliary anastomosis and T-tube placement was performed, and the CIT was 180 min. Enterococcus faecium was cultured from abdominal drainage fluid on POD 6 and treated with linezolid. He left the intensive care unit on POD 3 and was discharged on POD 28. At 18.2 months, the graft and recipient were alive, Doppler ultrasonography showed patent hepatic arterial and portal venous flow, and no biliary complication was diagnosed.

Donor and recipient characteristics are summarized in **Tables 1** and **2**. Perioperative and follow-up laboratory data for the four individual recipients are detailed in **Supplementary Tables S1**-**S4**.

**Table 1:** Donor characteristics and procedural data.

Variable	Donor 1	Donor 2
Gender	Male	Male
Age (years)	6	37
Diagnosis	TBI	HIE
CPR	No	Yes
High-dose vasopressors	No	No
Liver volume (cm³)	747	2062
University of Wisconsin Score	19	16
tDWIT (min)	18	28
fDWIT (min)	15	20
NRP time (min)	154	235
Mean NRP flow (L/min)	1.71	3.86

CPR, cardiopulmonary resuscitation; fDWIT, functional donor warm ischemia time; HIE, hypoxic-ischemic encephalopathy; NRP, normothermic regional perfusion; TBI, traumatic brain injury; tDWIT, total donor warm ischemia time.

**Table 2:** Recipient characteristics and outcomes.

Variable	Recipient 1	Recipient 2	Recipient 3	Recipient 4
Gender	Female	Female	Female	Male
Age	5 months	13 years	9 months	41 years
Diagnosis	BA	CC	BA	ALC
MELD/PELD	36	19	17	19
Child score	8	7	9	13
Graft	Left lateral segment	Right trisegment	Left lateral segment	Right trisegment
Graft weight (g)	140	510	430	1240
GRWR	2.55	1.46	3.91	2.18
Anhepatic time (min)	59	40	38	56
Operative time (min)	601	511	383	550
CIT (min)	269	160	87	180
Biliary reconstruction	Biliary-enteric	Biliary-enteric	Biliary-enteric	Duct-to-duct
EAD	Yes	No	No	No
PNF	No	No	No	No
Major postoperative complications	HAT, IVCT, IAI	IAI	ST	IAI
ICU stay (days)	3	2	2	3
Postoperative-LOS (days)	30	25	18	28
Graft survival	Yes	Yes	Yes	Yes
Recipient survival	Yes	Yes	Yes	Yes
Follow-up (months)	20.8	20.9	17.2	18.2

ALC, alcohol-related liver cirrhosis; BA, biliary atresia; CC, cryptogenic cirrhosis; CIT, cold ischemia time; EAD, early allograft dysfunction; GRWR, graft-to-recipient weight ratio; HAT, hepatic artery thrombosis; IAI, intra-abdominal infection; ICU, intensive care unit; IVCT, inferior vena cava thrombectomy; LOS, length of stay; MELD, Model for End-stage Liver Disease; PELD, Pediatric End-stage Liver Disease; PNF, primary nonfunction; ST, severe thrombocytopenia.

## 3 Discussion

This case series describes two applications of NRP-assisted *in situ* SLT from cDCD donors. The principal finding was that abdominal NRP provided sufficient physiologic support for hilar dissection, parenchymal transection, cold perfusion, graft retrieval, and subsequent transplantation of both split grafts. Both donors showed pH normalization and lactate clearance. All four recipients remained alive with functioning grafts at last follow-up, and observed complications were clinically manageable. These observations support technical feasibility and encouraging intermediate-term graft function, but they do not establish safety or comparative effectiveness.

To our knowledge, this is the first reported case series of *in situ* SLT from cDCD donors supported by abdominal NRP. The report addresses a practical limitation in deceased donor liver utilization. Traditional SLT, whether ex vivo or *in situ*, has generally been performed with hemodynamically stable DBD donors (6). Ex vivo splitting may prolong cold ischemia and increase the risk of bench-related injury, whereas *in situ* splitting depends on adequate donor stability and oxygenation. In DCD donors, these challenges are further exacerbated by unavoidable warm ischemia. Compared with conventional DCD procurement, NRP reduces the duration of warm ischemia between death declaration and cold perfusion while maintaining physiological organ perfusion throughout the technically demanding procurement procedure, thereby potentially mitigating ischemic injury.

The metabolic measurements provide objective context for graft acceptance. Lactate clearance and pH normalization are commonly considered alongside flow, hemodynamics, liver appearance, and other clinical information during NRP, but reported protocols and thresholds remain heterogeneous (13). The lactate reductions observed here were therefore interpreted as favorable trends rather than validated binary acceptance criteria. In addition, lactate sampled from the abdominal NRP circuit reflects the perfused abdominal compartment and cannot be attributed exclusively to the liver. Serial measurements, bile output, and standardized acceptance thresholds should be reported in future studies.

Several technical features likely contributed to the favorable outcomes observed here. First, femoral cannulation, systemic heparinization, and aortic balloon occlusion were established before WLST, allowing rapid NRP after death declaration while preventing cerebral reperfusion. This approach is consistent with international discussions and expert consensus on cDCD procurement, although implementation remains jurisdiction-specific and ethically sensitive (14, 15). Second, splitting was completed under warm oxygenated perfusion before cold perfusion and final vascular division, which may have preserved hemodynamic and metabolic conditions during parenchymal transection. Third, *in situ* splitting avoided additional cold bench time and preserved direct visualization for hemostasis and vascular control.

The recipient courses illustrate both feasibility and procedural risk. Recipient 1 met the Olthoff EAD criteria after HAT and required urgent thrombectomy, arterial bypass reconstruction, and inferior vena cava thrombectomy. Although the INR was elevated during the perioperative period, these abnormalities were closely associated with anticoagulation therapy. Following discontinuation of anticoagulation, the INR gradually improved and reached 1.34 at the last recorded follow-up. Sustained patency through 20.8 months is encouraging, but the event highlights the vulnerability of very small hepatic arteries (16, 17). Three recipients had culture-positive intra-abdominal infections, and Recipient 3 developed severe thrombocytopenia that resolved after TZP discontinuation without tacrolimus dose modification or platelet transfusion. The temporal relationship was compatible with TZP-associated thrombocytopenia (18, 19). Individualized immunosuppression allowed maintenance of a core agent while complications were treated, and no clinically treated acute rejection was recorded.

These findings should be interpreted within the following limitations. The procedures were performed at a high-volume center with established NRP and liver-splitting expertise and included only two donors and four recipients. Follow-up of 17.2 to 20.9 months is insufficient to characterize late vascular and biliary outcomes. The absence of PNF or graft loss therefore provides preliminary feasibility evidence rather than a safety estimate. Larger multicenter cohorts and registry-based comparisons will be needed before NRP-assisted *in situ* SLT from cDCD donors can be generalized beyond specialized centers.

## 4 Conclusion

NRP-assisted *in situ* SLT from cDCD donors was technically feasible in these two donation procedures and resulted in four functioning liver grafts for pediatric and adult recipients. The approach may broaden the practical use of cDCD livers for split transplantation, but it remains technically demanding and should be undertaken cautiously in centers with established NRP, cDCD procurement, and liver-splitting expertise. Further experience with longer follow-up will be required to define safety, selection criteria, and long-term outcomes.

## Data Availability

The original contributions presented in the study are included in the article/**Supplementary Material**. Further inquiries can be directed to the corresponding author/s.
