# Supplementary material for: Case Report: In situ split liver transplantation from controlled donation after circulatory death donors assisted by normothermic regional perfusion: a case series
Source: Front Immunol. 2026 Sep 22;17:1933200. doi: 10.3389/fimmu.2026.1933200 (PMC13638372; doi:10.3389/fimmu.2026.1933200)
Supplement: Supplementary file 1 [file DataSheet1.pdf]

CARE Checklist of information to include when writing a case report

CARE case report guidelines

(cc) BY-NC-ND

Topic	Item	Checklist item description	Reported on Line
Title	1	The diagnosis or intervention of primary focus followed by the words "case report"	1
Key Words	2	2 to 5 key words that identify diagnoses or interventions in this case report, including "case report"	33-34
Abstract (no references)	3a	Introduction: What is unique about this case and what does it add to the scientific literature?	11-16
	3b	Main symptoms and/or important clinical findings	17-22
	3c	The main diagnoses, therapeutic interventions, and outcomes	22-27
	3d	Conclusion—What is the main "take-away" lesson(s) from this case?	28-32
Introduction	4	One or two paragraphs summarizing why this case is unique (**may include references**)	48-70
Patient Information	5a	De-identified patient specific information	72-138
	5b	Primary concerns and symptoms of the patient	72-138
	5c	Medical, family, and psycho-social history including relevant genetic information	72-138
	5d	Relevant past interventions with outcomes	72-138
Clinical Findings	6	Describe significant physical examination (PE) and important clinical findings	72-138
Timeline	7	Historical and current information from this episode of care organized as a timeline	72-138
Diagnostic Assessment	8a	Diagnostic testing (such as PE, laboratory testing, imaging, surveys)	72-138
	8b	Diagnostic challenges (such as access to testing, financial, or cultural)	72-138
	8c	Diagnosis (including other diagnoses considered)	72-138
	8d	Prognosis (such as staging in oncology) where applicable	72-138
Therapeutic Intervention	9a	Types of therapeutic intervention (such as pharmacologic, surgical, preventive, self-care)	72-138
	9b	Administration of therapeutic intervention (such as dosage, strength, duration)	72-138
	9c	Changes in therapeutic intervention (with rationale)	72-138
Follow-up and Outcomes	10a	Clinician and patient-assessed outcomes (if available)	72-138
	10b	Important follow-up diagnostic and other test results	72-138
	10c	Intervention adherence and tolerability (How was this assessed?)	72-138
	10d	Adverse and unanticipated events	72-138
Discussion	11a	A scientific discussion of the strengths AND limitations associated with this case report	140-182
	11b	Discussion of the relevant medical literature **with references**	140-182
	11c	The scientific rationale for any conclusions (including assessment of possible causes)	140-182
	11d	The primary "take-away" lessons of this case report (without references) in a one paragraph conclusion	184-189
Patient Perspective	12	The patient should share their perspective in one to two paragraphs on the treatment(s) they received	-
Informed Consent	13	Did the patient give informed consent? Please provide if requested	Yes √ No
