# Supplementary material for: Case Report: In situ split liver transplantation from controlled donation after circulatory death donors assisted by normothermic regional perfusion: a case series
Source: Front Immunol. 2026 Sep 22;17:1933200. doi: 10.3389/fimmu.2026.1933200 (PMC13638372; doi:10.3389/fimmu.2026.1933200)
Supplement: Supplementary file 2 [file DataSheet2.docx]

**Table S1.** Perioperative and follow-up laboratory data for Recipient 1 (5-month-old female, left lateral segment graft, follow-up 20.8 months)

| **Variable** | **Pre-op** | **Day1** | **Day2** | **Day3** | **Day5** | **Day7** | **Discharge** | **Last follow-up** |
| --- | --- | --- | --- | --- | --- | --- | --- | --- |
| ALT(U/L) | 66.4 | 352.7 | 3692.1 | 613 | 453 | 76.1 | 5.9 | 18 |
| AST(U/L) | 205.7 | 1020.7 | 5711.7 | 696.7 | 178.9 | 27.1 | 17.8 | 36 |
| TBIL(umol/L) | 483.63 | 99.81 | 80.2 | 117.63 | 197.46 | 29.2 | 11.86 | 3.8 |
| DBIL(umol/L) | 327.68 | 40.95 | 50.05 | 83.9 | 161.79 | 15.88 | 3.19 | 1.3 |
| GGT(U/L) | 170.8 | 28.3 | 38.8 | 49.3 | 236.9 | 58.2 | 15.4 | 9 |
| ALP(U/L) | 1491.7 | 278.4 | 325.2 | 160.2 | 417.7 | 173.2 | 207.9 | 176 |
| INR | 2.47 | 2.02 | 1.68 | 4.61 | 1.7 | 1.69 | 1.87 | 1.34 |
| Cr(umol/L) | 19 | 29.7 | 30.2 | 29.8 | 17.2 | 25.9 | 21.1 | 16.2 |

Abbreviations: ALT, alanine aminotransferase; AST, aspartate aminotransferase; TBIL, total bilirubin; DBIL, direct bilirubin; GGT, gamma-glutamyl transferase; ALP, alkaline phosphatase; INR, international normalized ratio; Cr, creatinine; Pre-op, preoperative.

**Table S2.** Perioperative and follow-up laboratory data for Recipient 2 (13-year-old female, right trisegment graft, follow-up 20.9 months)

| **Variable** | **Pre-op** | **Day1** | **Day2** | **Day3** | **Day5** | **Day7** | **Discharge** | **Last follow-up** |
| --- | --- | --- | --- | --- | --- | --- | --- | --- |
| ALT(U/L) | 208.6 | 208.4 | 205 | 180 | 155.2 | 94.3 | 28.5 | 25.3 |
| AST(U/L) | 467.2 | 320 | 143.7 | 98.3 | 58.1 | 58.1 | 34.1 | 35.2 |
| TBIL(umol/L) | 93.63 | 56.23 | 27.51 | 37.22 | 26.92 | 20.19 | 7.37 | 8.5 |
| DBIL(umol/L) | 35.47 | 16 | 7.79 | 14.74 | 8.26 | 6.03 | 1.84 | 0 |
| GGT(U/L) | 69.4 | 46 | 33.5 | 83.7 | 83.1 | 55.9 | 38.2 | 10.5 |
| ALP(U/L) | 203.7 | 119.9 | 101.4 | 128.4 | 121.9 | 104.6 | 100.7 | 140 |
| INR | 1.75 | 1.64 | 1.4 | 1.26 | 1.27 | 1.37 | 1.31 | 1.16 |
| Cr(umol/L) | 84.9 | 101.4 | 92.2 | 71.6 | 69.9 | 99.6 | 54.2 | 76.4 |

**Table S3.** Perioperative and follow-up laboratory data for Recipient 3 (9-month-old female, left lateral segment graft, follow-up 17.2 months)

| **Variable** | **Pre-op** | **Day1** | **Day2** | **Day3** | **Day5** | **Day7** | **Discharge** | **Last follow-up** |
| --- | --- | --- | --- | --- | --- | --- | --- | --- |
| ALT(U/L) | 25.4 | 1436.6 | 829.9 | 776.6 | 326.2 | 243.8 | 90.3 | 39.1 |
| AST(U/L) | 75 | 952 | 371.5 | 247.9 | 96.3 | 73.2 | 76.7 | 53.5 |
| TBIL(umol/L) | 226.33 | 53.45 | 34.83 | 41.2 | 35.87 | 18.01 | 8.04 | 7.3 |
| DBIL(umol/L) | 156.6 | 30.13 | 18.98 | 26.63 | 26.5 | 7.9 | 1.86 | 1.4 |
| GGT(U/L) | 212.5 | 62 | 43.8 | 72.2 | 98.9 | 109.7 | 92.8 | 13.6 |
| ALP(U/L) | 662.7 | 210.5 | 125.7 | 166.4 | 303.5 | 388.8 | 321 | 265.9 |
| INR | 1.6 | 1.79 | 1.79 | 1.45 | 1.21 | 1 | 0.99 | 1.06 |
| Cr(umol/L) | 16.8 | 20.8 | 14.5 | 14.8 | 9.2 | 8.9 | 18.3 | 29.4 |

**Table S4.** Perioperative and follow-up laboratory data for Recipient 4 (41-year-old male, right trisegment graft, follow-up 18.2 months)

| **Variable** | **Pre-op** | **Day1** | **Day2** | **Day3** | **Day5** | **Day7** | **Discharge** | **Last follow-up** |
| --- | --- | --- | --- | --- | --- | --- | --- | --- |
| ALT(U/L) | 72.3 | 574.7 | 581.1 | 416.6 | 181.1 | 91.7 | 129.7 | 15.3 |
| AST(U/L) | 56.8 | 466.4 | 225.4 | 73 | 23.7 | 12.1 | 38.2 | 14.4 |
| TBIL(umol/L) | 253.02 | 61.56 | 35.43 | 34.31 | 46.35 | 36.02 | 13.27 | 15.43 |
| DBIL(umol/L) | 102.74 | 25.69 | 14.08 | 10.78 | 17.4 | 12.26 | 3.34 | 7.82 |
| GGT(U/L) | 28.9 | 32.8 | 30.6 | 29.8 | 42.4 | 37.7 | 117.1 | 18.7 |
| ALP(U/L) | 135.2 | 90.1 | 78.1 | 81.9 | 80.3 | 84.5 | 295.4 | 89.4 |
| INR | 2.38 | 1.6 | 1.11 | 0.94 | 1.02 | 0.91 | 0.9 | 1.08 |
| Cr(umol/L) | 41.6 | 47.4 | 36.8 | 35.7 | 33.4 | 26.7 | 49.3 | 54.4 |
